# Georeferencing the Natural History Museum's Chinese type collection: of plateaus, pagodas and plants

**DOI:** 10.3897/BDJ.8.e50503

**Published:** 2020-05-05

**Authors:** Krisztina Lohonya, Laurence Livermore, Malcolm G Penn

**Affiliations:** 1 The Natural History Museum, London, United Kingdom The Natural History Museum London United Kingdom

**Keywords:** natural history collections, botanical sheets, type specimens, digitisation, digitization, transcription, data cleaning, georeferencing, collectors, linked open data, sites, database, Chinese localities, herbaria, herbarium sheets

## Abstract

The digitising efforts of herbaria aim to increase access to and impact of scientific collections, by making the data digitally accessible to the global community. Digitising the NHMUK’s botanical collection of around 5.1 million specimens is an ongoing process, but the majority of the type collections have already been imaged. The Chinese type collection has also been transcribed; however, during the recent georeferencing process, we realised that much of the data had been transcribed incorrectly, particularly the locality information in which 80% of the collection contained errors. We discovered 154 specimens that were mistakenly filed in China. We corrected the mistakes from the previous transcription and georeferenced the collection which consists of 3,736 records. In this paper, we discuss the problems and errors we encountered during the georeferencing process, detailing why there were mistakes, what made the transcription harder than expected and what could have led to errors. We also give a short description about the Chinese language and its difference from European languages, leading to complex problems for georeferencing. We provide a brief guide on how to georeference a Chinese collection, avoiding errors and making the georeferencing process easier and faster.

## Introduction

The digitising efforts of herbaria aim to increase access to and impact of scientific collections by making the data digitally accessible to the global community. There are around 3,100 herbaria around the world, their collections collectively are around 390 million botanical specimens and that number is constantly growing (New York Botanical Garden 2020). These collections are a great source of evidence of the world’s diversity (Bebber et al. 2012). They can be used in identifying new specimens with DNA analysis, help the understanding of the similarities and differences between species and can help with investigating evolutionary and ecological changes (Lavoie 2013). The type collections are a fundamentally important part of the herbaria, as they are the primary source for identifying a plant specimen. Making these available online provides a crucial source of information for botanists around the world.

The Natural History Museum, London (NHMUK) launched a programme in 2014 to digitise the museum’s entire collection of 80 million specimens (https://www.nhm.ac.uk/our-science/our-work/digital-collections/digital-collections-programme.html). Approximately 5.1 million of these specimens are in the botany collection (Natural History Museum 2020). This collection includes scientifically and culturally important specimens collected from the 16th century up to the present day and the collection is still increasing (Penn et al. 2017). Within the NHM botanical collections, there are approximately 137,000 botanical type specimens which are of great importance to taxonomic and biodiversity research, as a type “is that element to which the name of a taxon is permanently attached” (Wiersema et al. 2011). The Natural History Museum’s botanical type collection was digitised and made available on JSTOR Global Plants platform as part of the Global Plants Initiative (GPI) (Ryan 2013), supported by the Andrew W. Mellon Foundation, but was not georeferenced at that time. The platform hosts more than two million high-resolution type specimens sourced from the major herbaria and this number continues to grow.

### Chinese type specimens

The NHMUK Chinese type collection was digitised and transcribed as part of the GPI project. The type specimen dataset contains 3,737 records, collected by more than 200 collectors, with geographical coverage from almost all of the provinces of China. The GPI project digitisation included taking a high quality digital image and transcribing labels, but did not include georeferencing (to give the specimens’ collection coordinates). The aim of the current project was to georeference the whole collection as close to an exact locality as possible, although we had to acknowledge that this would not be possible in some cases where the label is missing detailed locality information. A secondary aim was to identify as many unique site localities as possible to simplify and avoid duplication when georeferencing. Many collectors have collected multiple specimens at the same location, sometimes even returning to the same location at a later date. At some sites, multiple collectors have collected specimens, with some specifically visiting sites to follow an earlier collector’s collection routes. In such examples, we can use the same locality information for multiple collection events or specimen locations. It is important to identify these sites, as examples of repeated collecting over long periods of time are comparatively rare and these data are crucial for projects that use specimens to understand diversity and evolutionary changes.

During the process of georeferencing, we found that the existing transcribed label data did not accurately represent what was on the specimen label. Careful checking of the label information showed that up to 80% of the locality data transcribed was inaccurate, therefore the label information had to be checked for each specimen and re-transcribed where necessary.

### Transliterating Chinese

One of the challenges in transcribing these specimens was the transliteration (and occasionally translation) of Chinese. As a language completely different from the European languages and writing systems, transliteration is not an easy task. Transliteration (romanisation) of Chinese has changed a lot in the last couple of hundred years, especially in the 20th century, covering the time period when many of the specimens in this project were collected. While it is beyond the scope of this paper to give a comprehensive overview of Chinese translation and romanisation, we hope to give the reader a short primer and where they can find more information.

#### Chinese Romanisation Systems

Romanisation systems translate the characters and spoken word into Latin letters. There is now a standardised romanisation system for Mandarin Chinese, Cantonese, Hokkien and other Chinese dialects, but back in the early 19th century, different countries had their own systems. Legeza (Legeza 1968, Legeza 1969) states fifty different romanisations. The Wade-Giles romanisation system for Mandarin Chinese was first developed by Thomas Wade in the mid-19th century, it was completed by Herbert A. Giles and then published in Giles’s Chinese–English Dictionary in 1892. The Wade-Giles system was based on the Beijing-dialect, which is the base for the current Standard Mandarin Chinese.

The French E.F.E.O. (École française d'Extrême-Orient) developed a romanisation system in 1902. It was similar to the Wade-Giles system and it was used by the French-speaking world until the mid-20th century. The EFEO is quite similar to how Chinese was transcribed by French missionaries in the late 17th to early 19th centuries (see Du Halde 1736).

The Yale romanisation system was developed in 1943 by the Yale sinologist George Kennedy, mainly as a course to teach Chinese to American soldiers. It is based on Mandarin Chinese but transcribes the Chinese sounds to emulate how English speakers would form them, making it easier to pronounce for English speakers. It is uncommon on our specimen labels, but is used in books that we have used to find historical localities.

Before these systematic romanisation systems, explorers, botanists and missionaries used Nanjing dialect-based romanisation or their own personal systems. It is important to mark the differences between romanisation systems and what their origin was, as the Beijing dialect is the basis of Standard Mandarin Chinese, which is the official language of the People’s Republic of China (Order of the President of the People's Republic of China 2000). These days, official Standard Mandarin Chinese is taught in schools for children from areas where the local dialect differs from the Beijing dialect and, in international schools, it is also the Beijing dialect that is commonly taught. Generally, in the northern area of China, the dialects are similar enough to one another that they and the Beijing dialect can be mutually understood. In other parts of China, especially in the South, the dialects can differ greatly (Ramsey 1987), hence romanisation systems that are based on different dialects, can differ in their end results. Examples of different romanisations are given in Table 1.

To compare romanisation systems and to help with transliteration from one system to pinyin, there are a number or sources and tables available online, e.g. Wikipedia, Pinyin.info , as well as books or publications such as *Chinese Romanization Systems: IPA Transliteration* (Shibles 1994). Pinyin (Hanyu Pinyin) is the official romanisation system for Standard Chinese (Mandarin) using Latin letters.

## Methodology

It is hard to codify the entire transcription and georeferencing process, as many aspects are based on experience, but we have tried to describe the general steps and some significant considerations.

It is essential that the entire transcription and georeferencing process follows a set of rules and guidelines in order to reduce errors and enforce consistency. We followed the NHMUK’s georeferencing protocols and geographical standards (Penn et al. 2017), that provide instructions on how to mark missing or uncertain information in the correct fields and how to georeference general spatial locations (Blagoderov et al. 2017).

We have provided our table of spelling variations of Chinese provinces in Suppl. material 4 compiled from Law 2015, as well as a table of useful links in Suppl. material 6 and a list of the often used localities in Suppl. material 5.

### Transcription methodology

Handwritten labels are harder to interpret, so marking uncertainties on the transcription is helpful for subsequent users of the data. In case of uncertainty by the first transcriber, someone else can look at the label or georeferencers themselves can take a second look on the label.

Prior to transcription, we agreed on and used a set of formal abbreviations, often historical and based in Latin, for sections that cannot be transcribed as the information is missing from the label. This is fairly common in natural history collections (Table 2).

Blank fields should only appear for records which have not been transcribed yet. If the transcriber is uncertain of the information, they can indicate that with brackets, square brackets or dots, whichever the institutes prefers to use in their collection system (Groom et al. 2019). We used the following indications (the bold words in Table 2), we also used “Illegible” for illegible collector names and “[illegible]” for illegible locality information. In case of uncertainty, we used “[word ?]”, indicating that we are unsure of the transcription of the word (to the extent that we could not say whether it is a locality, collector etc).

#### Collector and date

We started the transcription process with the collector and the date. Chinese romanisation systems were vastly different in the past and based on the collector’s mother tongue. Determining the collector and their background was the first step in identifying which romanisation system to use when georeferencing the collecting locality. In cases where the collector is a Chinese national, we needed to consider in which part of China they collected and when and where they were educated and employed. For example, there were a number of Chinese collectors working for Harvard University. Those labels have the Chinese locality’s names usually by the Wade-Giles Romanisation systems.

#### Collection date and collection number

**Figure 1.**
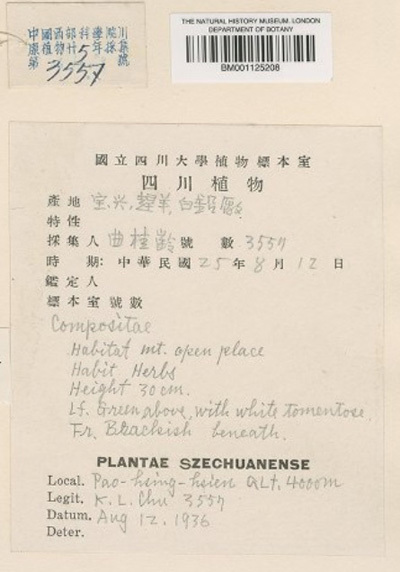
Specimen BM001125208 - Example of a label written in Chinese and English with more information in Chinese than in English.

**Figure 2.**
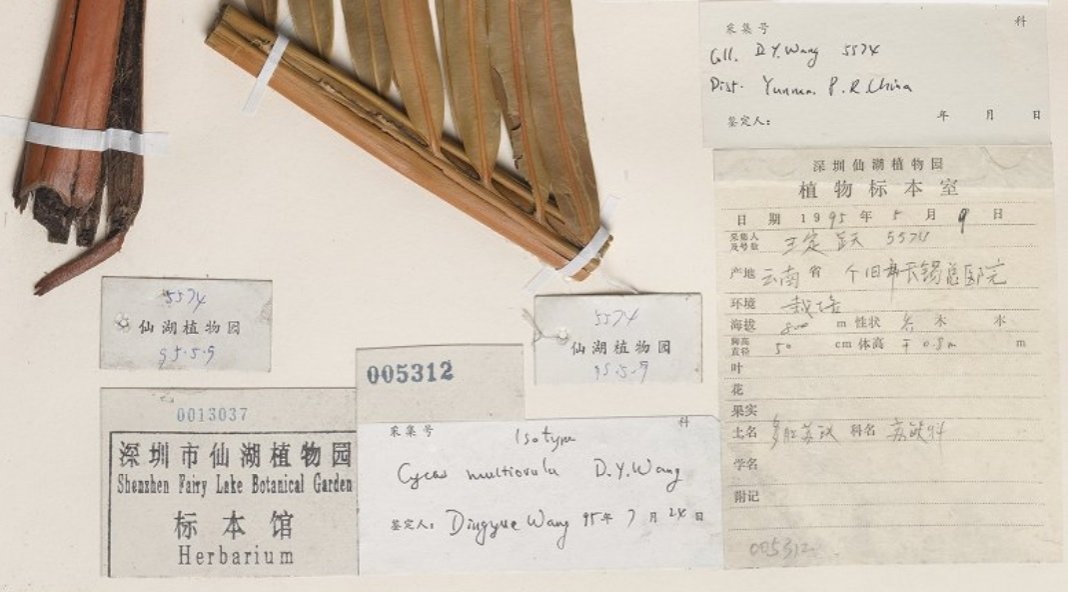
Specimen BM000796373 - Example of a Chinese label with the English locality transcribed on one of the determination labels.

**Figure 3.**
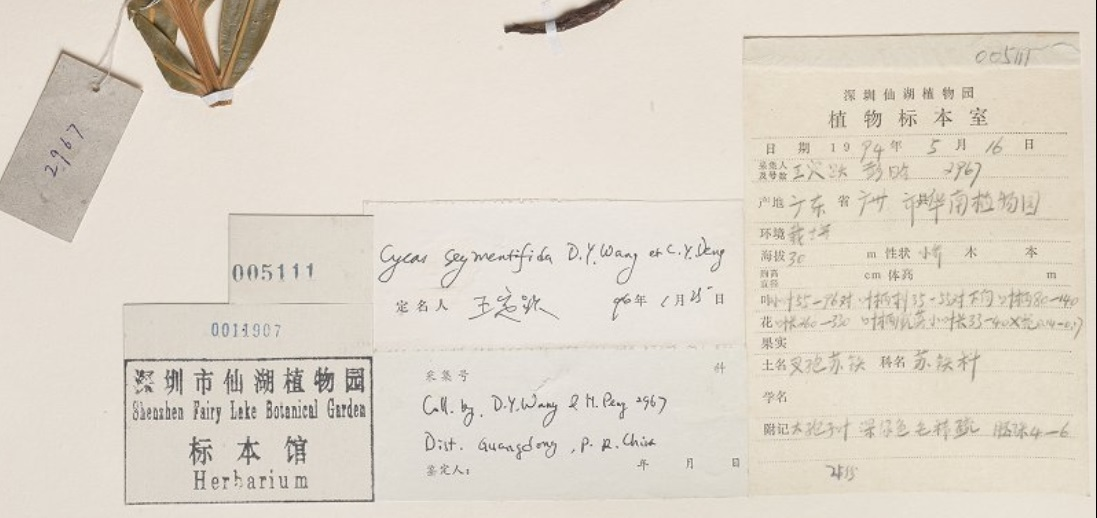
Specimen BM001047709 - Example of a Chinese label, with English locality transcribed on one of the determination labels.

The next step was transcribing collection date and collection number. We frequently encountered partial dates, for example: only month and year, only year or, occasionally, a date range in years. When the collection date was written as “summer of”, we transcribed the year to the correct field and the rest of the general information in an unstructured notes field. Date can also help when georeferencing certain areas, for example Manchuria or Taiwan: both were ruled by Japan for parts of the 20th century. If we find a collection label during the period of Japanese control, then the locality will most likely have the Japanese names. Similarly in Manchuria, which changed hands through the centuries, the locality names can be in Chinese, Japanese and Russian. The collection date can also determine the most likely Romanisation system. Newer collections (collected around and after the 1950s), collected by Chinese nationals will have the official pinyin name on the label. There are frequently two labels on the sheet, one in English and one in Chinese, but the Chinese label usually has more information than the English one, as seen in Fig. 1. You can see an example below with only a Chinese label and partial locality information on the determination label (Figs 2, 3).

#### Locality

Properly transcribing the label’s locality is of the utmost importance when georeferencing. Due to the complexity and the high number of romanisation systems for Chinese, even small variations such as single quotes in the middle of the word or accents on the top of the vowels are important for translating and transliterating them; missing out a single quote in the middle of the word can completely change the results when a georeferencer is translating the earlier romanised name to modern pinyin (Table 3).

Transcription of the locality therefore needs to be not only word by word, but character by character, to avoid subsequent errors during georeferencing. To correctly find localities, we need as much information as possible. Sometimes the label is vague and may only mention the exact locality and omit country and province information; this makes georeferencing much harder. Knowing the collector and the date can allow a georeferencer to identify the area in question by looking at other labels from the collector, where they were collecting around the time when the label in question was written.

### Collectors

As supplementary data, we created a dataset about the collectors from whom we have specimens in the Museum’s Chinese type collection (Suppl. material 1). This includes a small number of collectors who turned out not to have collected in China, but whose collections had been mistakenly filed in the Chinese geographical region. Since their collection was also georeferenced as part of this project, we have kept them in the dataset. For all the collectors, their Global Plants Collector profile is included where available, as well as their Wikidata biography page, for example, the page for Henry Fletcher Hance (Wikidata Contributors 2019) . From the 265 collectors, 180 of them had an existing Wikidata profile. For 37 collectors, we created their page, based on biographical information. For the remaining 48 collectors, the biographic information was too limited to be able to create a Wikidata page.

While transcribing, we checked the collector’s active working years and possible expedition descriptions to correctly identify them. We also compared label information to their biography information on Global Plants. For collectors with no Global Plants profile, we tried to find biographic information via Google or other sources. For Chinese collectors, *Plants of China* (Hong and Blackmore 2015) was especially helpful. Access is limited on Google books, but there is a copy of it in the NHMUK’s library. It provided a list of Chinese collectors, with names in pinyin, Chinese characters, variants of their names, birth and death dates and sometimes biographic information.

In botanical collections, we also have specimens collected by people who were not botanists by profession, but collected plants and sent them to other botanists or institutes. These collectors were biologists, entomologists or, quite often, other professions such as missionaries, doctors and surgeons. For these collectors, other sources were useful to find biographic information, depending on their affiliation. For missionaries, the best sources for biographic information are documents issued by the corresponding church about their missionary activities. Obituaries are also a good source, as they often describe the deceased person’s life in detail.

Our criteria for creating Wikidata profiles were based on having the following information available: full name or family name with initials at least; one or more categories of biographic information with a reference, for example, birth date, birth place, institute they worked for or graduated at etc.

Unfortunately for certain collectors, we only had limited or ambiguous information on when they were definitely alive or who they were. For these collectors, we were unable to create a profile at this point.

### Georeferencing methodology

**Figure 4.**
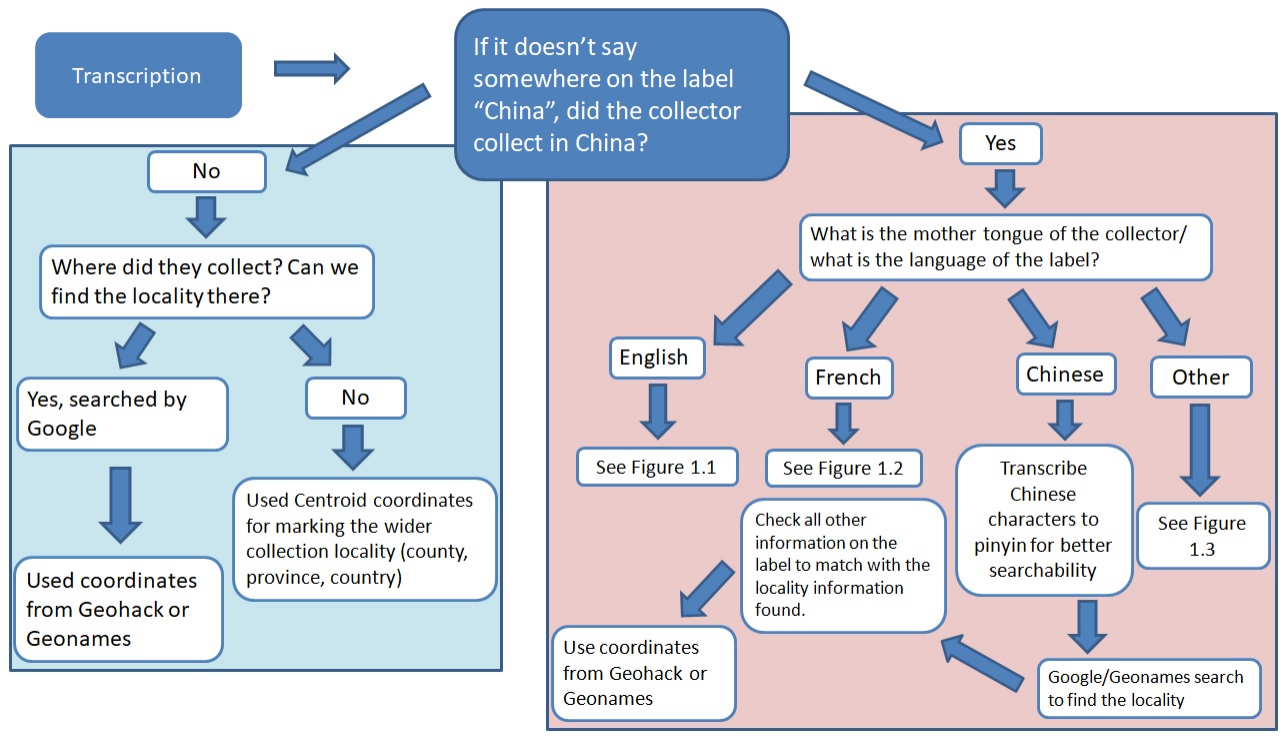
Flow diagram on how to approach labels in different languages.

Following transcription, the next step is georeferencing. In this project, we followed a method shown in the flow diagrams below (Fig. 4). Although we always tried to find coordinates for the exact locality, this was sometimes not possible due to incomplete labels. In situations like these, we used centroid coordinates for the closest locality we could confirm, such as a country or a province. The protocol we used can be found in the article by Blagoderov et al. 2017 (Supplementary material 2). These centroid coordinates were mathematically defined using ArcGIS, using the Geometry tool, in ArcMap, ArcGIS. ESRI, ArcGIS Software.

During the georeferencing process, we found that some of the ‘Chinese’ specimens are likely to be from different geographical regions. Dealing with an area with a long history of border changes, it is possible that the collection locality was part of China at the time of collection. For mountainous or remote areas, like Tibet, it is also possible that the collector(s) were unaware of the border crossing. However, mistakes can happen at any time and it occasionally happens that a sheet was mistakenly filed in a certain geographic region when, in fact, it belongs to a different region, sometimes not even bordering the one we are working with. Therefore, we did our best to carry out our own investigations, to make sure we did not spend time looking for a locality in a completely different country. When in doubt, we searched the collector’s biography on JSTOR Global Plants, to see which countries they worked in and compared this to our label information to determine where we should look to find the correct collection locality.

When the specimen had a Chinese label, we transcribed the Chinese characters into pinyin, to make the search easier, then used Google to locate the correct collection site.

**Figure 5.**
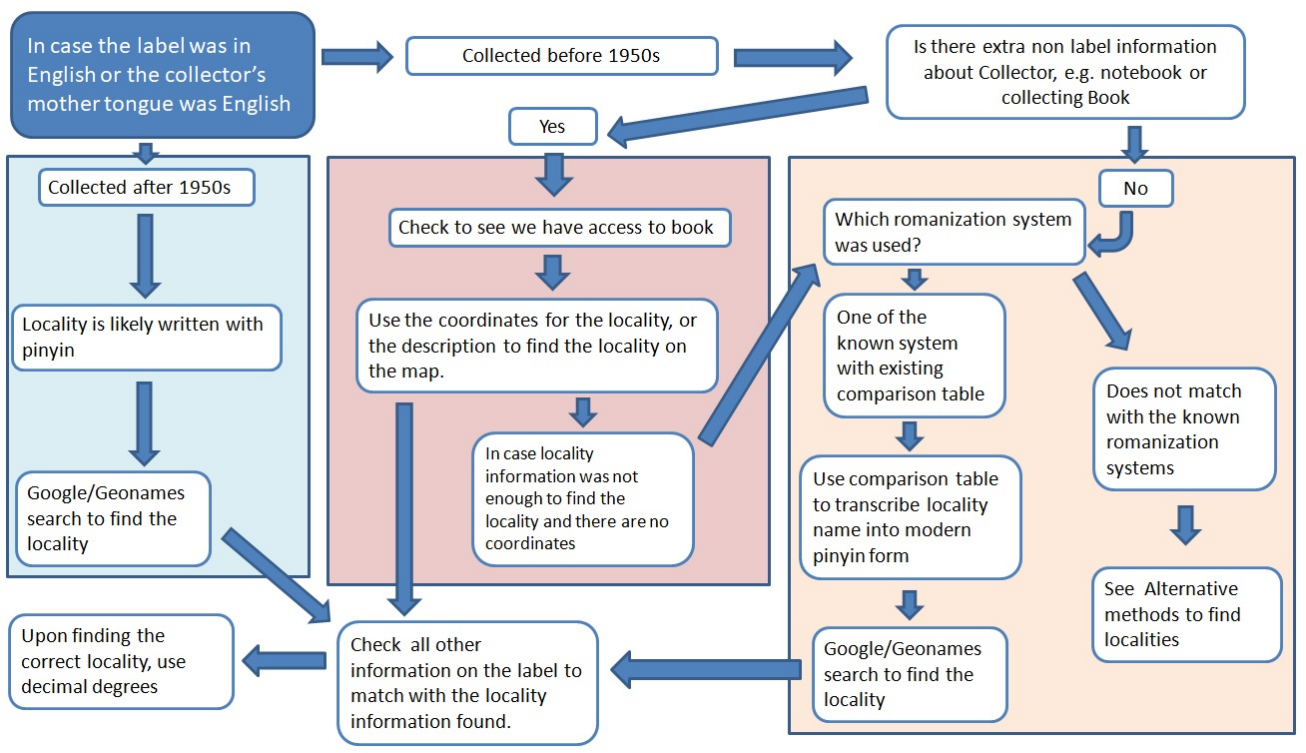
Flow diagram on how to approach labels written in English.

Where labels were in English, we followed the procedure show in Fig. 5.

If the specimen were collected before the 1950s, the first step was to check if the collector has published a book about their travels or experiences or whether this was covered in any other publication. JSTOR’s database is a good source for these publications, as well as archive.org and occasionally Google books. Publications about collecting activities often provide multiple spellings of collection sites for easier identification and, where available, coordinates as well. In books written by collectors themselves, we often find descriptions of collection sites and how they got there and therefore, following the description of the route on Google maps, we can often positively identify the collection locality.

When there was no book/publication available or the locality was not mentioned, we needed to identify the romanisation system used by the collector. This was a manual process using the commonly-used systems in Table 1, but we also used less common, usually outdated systems described in Legeza (1968), Legeza (1969).

The most complex and problematic sites were the ones where there was no book or publication available and the locality name did not match any of the commonly-used romanisation systems. For these labels, please find the suggested procedure in the Alternative sources to find localities section.

It is worth mentioning that by familiarising ourselves at the start of the project with the most common or relevant geographic terms on the labels (i.e. city, river, mountain, valley etc.), we can more easily determine what part of the label has the locality information and name. We used a pre-made list as a ‘cheatsheet’, including not only the Mandarin Chinese words for the English equivalent, but the same words’ spellings under different romanisation systems and/or in different languages (e.g. French).

For French labels, follow Figure 4.1, but use the following steps if the answer for “Is there extra non label information about Collector” is "No":

1. Check a EFEO : pinyin comparison chart (easily accessible via Google)
2. Use comparison table to transcribe locality name into modern pinyin form
3. Google/Geonames search to find the locality

If the collection pre-dates the EFEO system:

1. EFEO comparison table is still usable, as the system was based on the transcription of French missionaries

If none of the methods helps, see Alternative methods to find localities paragraph.

For labels in languages other than English, French or Chinese, the flow diagram on Figure 4.1 can be followed and, if there is no extra non-label information about the Collector, see the section on alternative methods.

As we had labels written in various languages, in order to make work easier, we created a reference table with the most frequent generic localities in various languages (Table 4).

#### Alternative methods to find localities

When simple map and google searches, publications and romanisation tables did not bring results, we had to find alternative methods to look for the localities.

**Figure 6.**
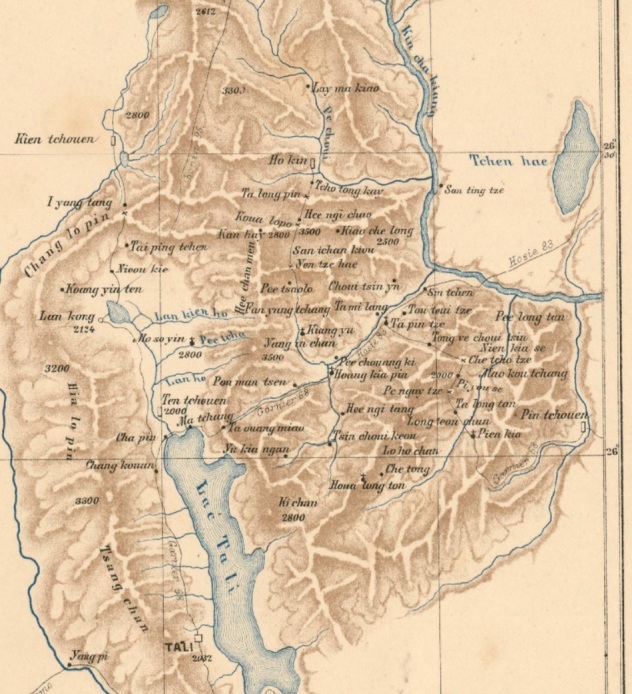
A map of the Yunnan region by Père Jean-Marie Delavay (1898).

Generally old maps (a good source is David Rumsey’s Map Collection); mapping websites (for example, Geonames, geoview.info, Peakery); publications; Google and Wikipedia are good sources of locality information. We used a range of publications and books, including Western travel journals of visits to China, for example, Robert Fortune's *A journey to the tea countries of China*, Frank Kingdon-Ward’s *The land of the blue poppy* (Fortune 1852, Kingdon-Ward 1913), which can be essential sources of localities, especially if written by collectors themselves. These are their first-hand experiences, providing detailed descriptions of the localities they visited, usually describing their routes to collecting sites, which can be quite informative, even if the surroundings changed or are not marked on maps. With very detailed accounts, geographic coordinates can be acquired this way. Many of these collectors actually used hand-drawn maps, where the localities have the same name as on their labels. Even if the locality is not marked on modern maps or the name has changed, we can still find the approximate location by using these hand-drawn maps. Occasionally, coordinates on these maps may not be reliable, but since the maps usually include topographic features, this helps us find the right location (for example, the map by Delavay, Fig. 6). However, even when books were written by someone else, they can contain vital locality information. The best approach in these situations was to search online for the locality’s name, exactly as written on the label, with extra words, such as “china”, the collector’s name and any other locality indicator. Usually, this would identify a book on archive.org or google books which included these terms. Sometimes, it was a paragraph written by someone else, mentioning that that specific person collected some plants somewhere. Some were descriptions from other expeditions to the same location. When two or more collectors worked in the same area, if at least one drew a map and published it, it is a good assumption that we will find the localities of others as well, who collected in the area around the same time. For example, Francis Kingdon-Ward published many maps along with his books about his collecting activities and these proved a useful source for other collector’s localities as well.

Collectors often used distinct features of the locality such as ancient buildings, religious temples etc. as physical references. If these sites do not exist anymore, the most unique feature of the area is lost. For finding exact locations in China, tourism information websites can also sometimes be useful. During the cultural revolution, many ancient sites and cultural buildings were destroyed, although there is an increase in creating memorial sites (plate or board with information about the monument existed on the spot before) or creating a page or article about it and sharing online. This usually applies to old Chinese sites and monuments, for example, the ancient pagoda near the sea in Amoy/Xiamen, which has since been destroyed, but appears on some labels as a collection locality (for detailed account about finding the exact location of the pagoda, see the Discussion section).

Finding mountains peaks and passes in the Himalayas can be difficult, unless we find travel blogs from modern hikers. The mountain passes are usually called by the same local name as they used to be, even if this is not marked on maps. These internet blogs occasionally give detailed accounts, photographs, mentions of distinct topographic features and occasionally coordinates attached to photographs. There are blogs written by people who chose to retrace a collector’s travel route, for example, " In the footsteps of Joseph Rock" (W 2020).

When the collection was made by a French speaking person before the development of the EFEO system, the EFEO system can still be used as a guide, but Biot’s Dictionnaire des noms… (Biot 1842) is a great source for Chinese localities. It describes all sorts of localities: cities; villages; administrative divisions; and so on, including the Chinese characters and a description where the locality in question is found. It can also be used as a guideline to how certain Chinese sounds were written down by the French missionaries.

When we encountered collectors whose system did not match any romanisation comparison charts and where no publication documented their collecting activities, the best approach was to stop after transcription. When the transcription was complete for the entire Chinese collection, we grouped together the specimens collected by the same collector to review the labels. As collectors usually collected multiple specimens at the same place and time, some labels can contain more information than others. Grouping the collection by collectors and then organising by collection time and date, could also help infer routes of travel. Where we found some localities, plotting these data points could help narrow down nearby areas from where those collectors were most likely to have collected. These conclusions were based on collection information from the rest of the collection, collection of other herbaria published on Global Plants and biographical information. This method also helped to exclude localities which matched the general information on the label, but were unlikely based on other data. For example, we were able to rule out some localities where it became apparent that the collector could not have travelled that distance in the period of time between confirmed collection events. It is also important to note, that this method was only used to exclude locations from the “possible collection locality” list, as there could be several other settlements, rivers or other locations bearing the same name and not present on the maps we used to georeference.

Examples for georeferencing a collection include: specimen BM001044167 where due to correct transcription and careful research, the collection locality could be found; and specimen BM001066167 where the exact locality could not be found. It may be possible to determine the exact locality, but we were unable to do so in this project.

This type of locality research usually took between 5-15 minutes, with occasional locations taking longer. After a couple of minutes, it was usually possible to determine if there was a likely positive result and decide to stop or continue checking. Sometimes, we exhausted all avenues of enquiry or determined we had a false lead, while sometimes the research required to correctly determine localities would require much more than 15 minutes to determine the exact locality, which was beyond the scope of this project. We are hoping that some of these indeterminate localities can be resolved in the future as more collections and researchers work on georeferencing specimens and make this information available online.

### Example 1: BM001044167

The label says “Herb. H. F. Hance”, meaning it is from the herbarium of H. F. Hance. H. F. Hance is Henry Fletcher Hance and it says “Ipse Legit” on the bottom of the label, meaning “collected himself”. The collection date is 1866. If we check Hance’s biography, in 1866 he was in Guangdong Province in China as a Vice-consul.

The verbatim information is: “Ad coenobium buddhisticum Filoi tsz secus fl. North River, prov. Cantoniensis.”

As the collection date is 1866, we cannot use Wade-Giles or any other known romanisation chart. What we do know that “prov. Cantoniensis” is approximately the current Guangdong Province (Wikimedia Foundation, Inc. 2019). “secus fl. North River” means that it is “near North River” (= Bei river). (See the river map)

In the The Journal Of Botany British And Foreign Vol-v (Hance 1867), there is a paragraph about the place in the “On Liquidambar Formosana, Hance” section by H. F. Hance.

“"in an excursion I made with Mr. Sampson up the North River to the Tsing-yune Pass, about 120 miles above Canton, in the magnificent dense woods encompassing the renowned Buddhist monastery of Filoi-tsz"

Tsing-yune Pass = Qingyuan Pass, which is near Qingyuan city towards Canton (Guangzhou) on the North River. At that time, it was common to travel on the rivers in China. Hance was stationed in Guangzhou at the time. He also mentions Mr. Sampson, who is Theophilus G. Sampson. Many of his collected specimens were given to H. F. Hance and the Filoi-tsz Buddhist monastery often appears for specimens he collected.

There is another good description about the area mentioning the same sites in the book *Through China with a camera* by John Thomson (Thomson 1899).

In that direction, away from Guangzhou, on the North River, near Qingyuan, there is one well-known Buddhist temple, the Feilai Temple (飞来寺). In the spelling Hance used, the “tsz” stands for “寺” (pinyin: sì or si4), meaning “temple”.

At the end, we double checked that all the information matches. The Feilai Temple is in Guangdong Province, on the North River. From the paragraph written by H. F. Hance, we know he travelled there in 1864 and also that, in 1866, he was living in Guangzhou. The Feilai Temple was built in AD 520 and it still stands. Therefore, we concluded that the “Filoi-tsz” on the label is indeed the Feilai Temple.

### Example 2: BM001066167

There is no collection date, but a collection event code: 9158 and a collector's name: Henry.

The verbatim (collection locality) information is: Yunnan, Man-mei; alt. 7000'.

The collector could be Benjamin Couch Henry or Augustine Henry. Augustine Henry is more likely, as he indeed collected in Yunnan Province. For more certainty, we looked at the historical literature, including the Protologue as well. Unfortunately, there is no first name mentioned. We then searched for duplicates of the type, with the same species, collection number and locality. Fortunately, there were three other specimens and the collector marked “A Henry” on all three. Therefore, it is highly likely that the collector is Augustine Henry (1857 – 1930). He was English speaking and living around the time when the Wade-Giles romanisation system was developed. We do not have a collection date, but the Protologue was published in 1898, therefore, the collection has to be earlier. We cannot determine what romanisation system A. Henry used, but we can exclude the EFEO, as he was not French speaking and the Gwoyeu Romatzyh, which was developed in the 1920s. Using a romanisation comparison chart (Pinyin.info), we can see that “Man” and “mei” is written the same way by all systems. It is safe to assume that we can search for the name “Manmei” without transliteration. The dash/hyphen is not used in the Chinese language, only in the Western romanisation systems, while the current pinyin system does not use it either.

Using the Geonames search engine for “Manmei” in Yunnan Province, we get five records, three of them an exact match to our name on the label. From the Protologue and the duplicates, we get the extra locality information “S. of Red River” or “Mts. S. of Red River”. From the three records, two of them are South of the Red River and we can reason that 漫美 (Manmei) is the most likely, as it is closest to the south bank of the Red River. However, we cannot be certain and, without the exact date he visited the location, at this point, we cannot identify the locality with absolute certainty.

## Results

**Figure 7.**
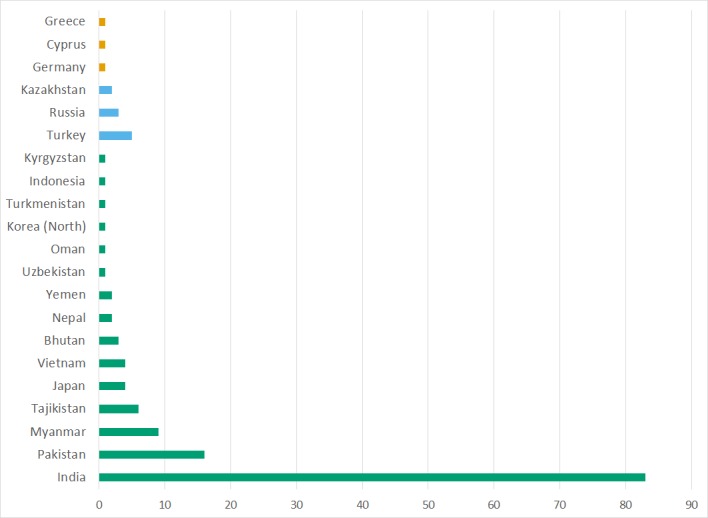
Number of specimens incorrectly recorded from China group by country and region (Europe = orange, Asia/Europe = blue, Asia = green).

Amongst the 3,736 records filed in the Chinese geographical region, we found records from 24 other countries. Some were the result of border changes and disputed territories like Tibet and Manchuria, while others were due to transcription errors. Of the 3,736 records, 3,582 records were from China; the country distribution of the remaining 154 is shown in Fig. 7Suppl. material 3) . The greatest percentage is India and Pakistan, largely due to the change of the border over the Tibetan territory. We also have nine records from Myanmar, from collectors who collected near the border in Yunnan.

The 3,588 records collected in China are from 34 provinces including the Hong Kong special administrative region. A total of 50% of the records comes from three provinces: Sichuan (658), Tibet (446) and Yunnan (720 records) (Fig. 10, Suppl. material 2). These provinces were usually considered the most biodiverse provinces (Stepp et al. 2002), many collectors leading years-long expeditions specifically exploring the Tibetan Plateau, Sichuan and Yunnan. George Forrest (a Scottish botanist), from whom we have 416 specimens in the Chinese collection, collected in China for 28 years covering all three provinces mentioned above. Augustine Henry, a British-born Irish plantsman and sinologist undertook a 4-year expedition, mainly covering Hubei, Sichuan and Yunnan. Ernest Henry Wilson, a British plant collector and explorer, had several expeditions to China, mainly covering Hubei and Sichuan. Frank Ludlow was a British naturalist and officer of the British Mission, stationed in Lhasa. His collections cover the Tibetan Plateau, mainly the Tibet Province of China and Arunachal Pradesh state of India (which is a bordering state with Tibet).

The collection is the result of the efforts of 265 collectors from the mid 18th century to the 2010s. Nineteen of the collectors did not collect in China but collected some of the 155 specimens collected outside China.

**Figure 8.**
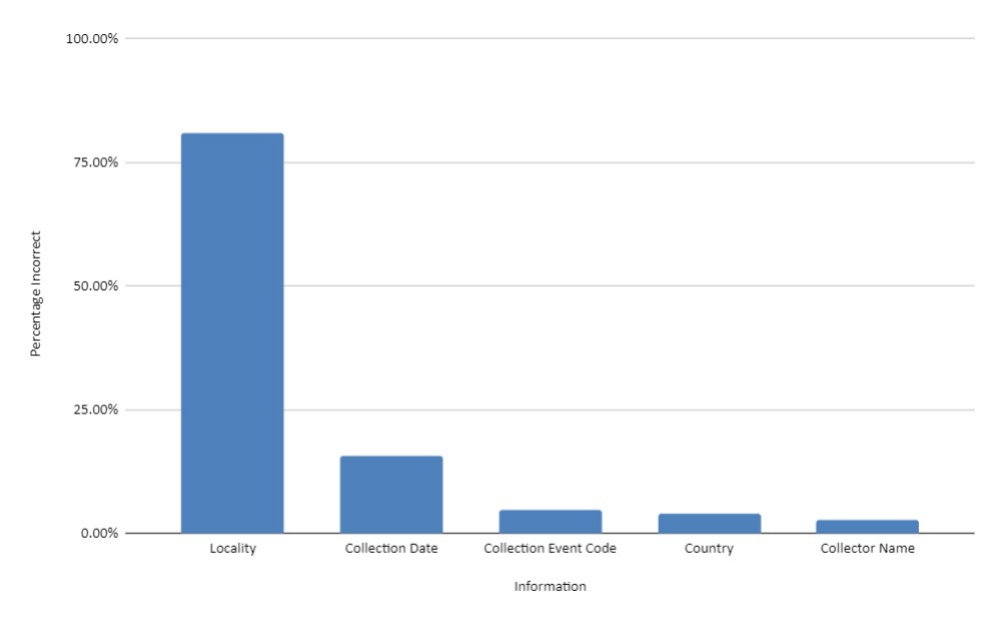
Error rates in transcription.

In terms of the accuracy of the original transcriptions, the lowest error rate was for Collector name, Collection Event Code (Collection number) and the Country (Fig. 8). For the collector, occasionally the wrong collector was identified, such as people with the same family name or where the wrong person was identified as “collector” from the label. In botany, it is often the case that people collected on their travels or during work overseas on the botanist’s behalf, then shipped the collected material to them. On the labels, sometimes it says “collected for” and then a name. This person was not the one who collected the specimen, but rather than the person who commissioned the collection.

Errors in the collection event code were usually transposed numbers, missed collection number or catalogue number incorrectly identified as collection number. This included Wallich’s catalogue numbers identified as collection numbers.

Country mistakes were mainly due to border changes or to disputed territories.

**Figure 9.**
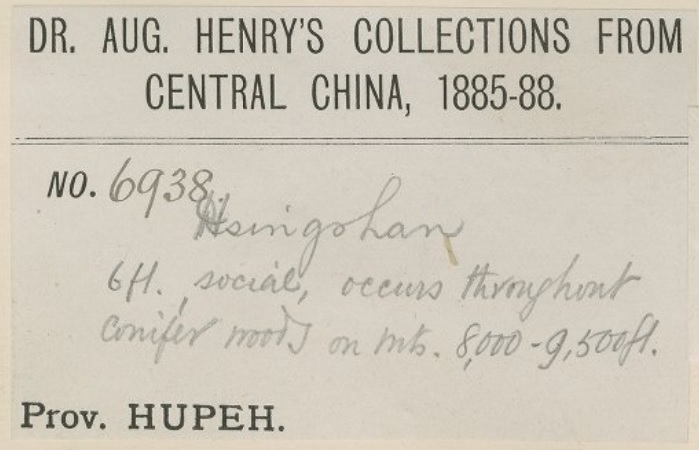
Specimen BM000959201 - The expedition and its date can be seen on the pre-printed label, but the collection number and collection locality is handwritten. In the original transcription, the locality information was missed. It was a frequent occurence for Augustine Henry's specimens to not have their collection date transcribed (1885-88) or to have partially transcribed locality information.

**Figure 10.**
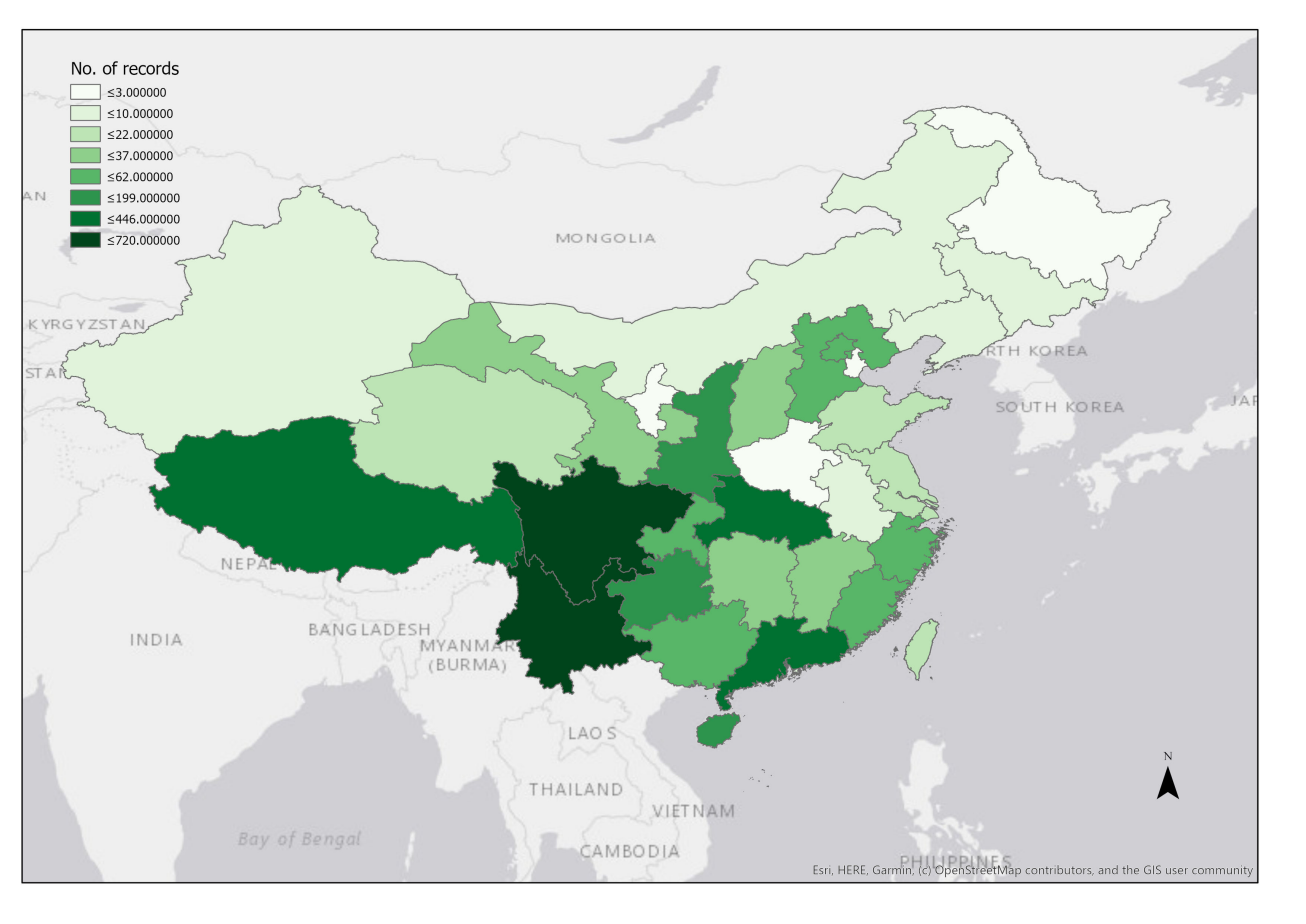
Chloropleth map showing relative frequency of NHMUK Chinese type specimens collected from Chinese provinces.

The transcription error rate was higher in Collection Dates. The main cause could have been that transcribers were unaware of the use of pre-printed labels in the botanical collection. For larger collection expeditions, some collectors had pre-printed labels, where the name of the area/country was printed and also the years of the expedition (see Fig. 9). Sometimes, they wrote the exact date (day/month) by hand or just did not write exact date at all. Many of these labels were not transcribed fully and the printed information was often not transcribed. Figure 9 shows a typical label from Augustine Henry. The collection date in that case is the pre-printed “1885-88”. Occasionally, we can find exact dates for the collections in the Protologue or publications, but from the label alone, that is the only collection date we have.

The highest percentage of errors were in the Locality. The mistakes were mainly missed information, as previously mentioned, with pre-printed labels. In addition, Country names and/or provinces were often not transcribed (sometimes where the exact locality was transcribed, but not the country or province to situate it correctly) and altitude markings were frequently absent from the transcribed data.

Frequently, where the locality information was not transcribed, the field was not marked in any way, for example, with [...] or [illegible], to show whether locality information existed, but was illegible. Similarly, if there was no locality information, the field should have been marked as “sino loco” or an accepted abbreviation of the expression.

## Discussion

**Figure 11.**
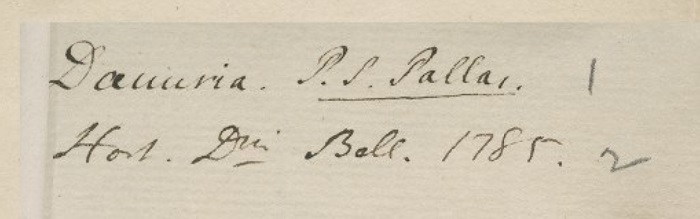
Specimen BM000554710 - "P. S. Pallas" is Peter (Pyotr) Simon von Pallas. He did not collect in China, but collected in the territory of the Russian Federation. The locality "Dahuria" is in Russia.

**Figure 12.**
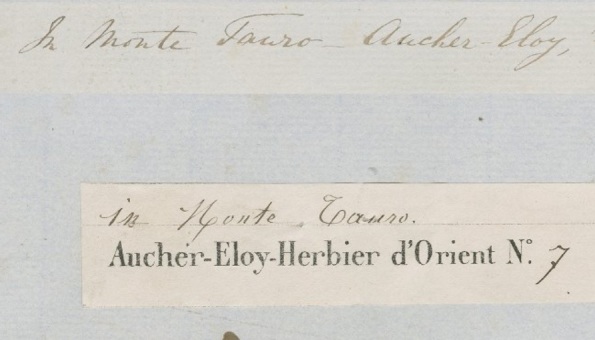
Specimen BM000559554 - The only locality information is "Monte Tauro". We searched for the collector (Aucher-Eloy) and found he did not collect in China, but collected in the Mediterranean. Monte Tauro is in Turkey, where he did collect.

**Figure 13.**
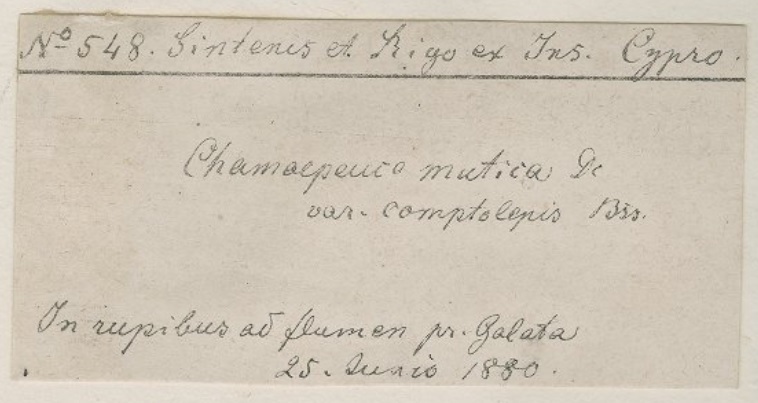
Specimen BM000996109 - The collectors are Paul Ernst Emil Sintenis and Gregorio Rigo, but neither of them collected in China. This helped us to determine that "Ins. Cypro" (Insulam Cypro) refers to the island of Cyprus. Both Sintenis and Rigo collected there.

In this project, we worked with a pre-transcribed dataset so the data required for the georeferencing was already present - at least in theory; the main task was to find the exact location of the localities transcribed. The pre-transcribed data, depending on the corresponding label quality and the error rate, held associated information that made georeferencing easier. For example, knowing the collector name reduces the number of possible countries a specimen could have been collected from and the possible timeframes. This can help with spotting mistakes, for example, when the specimen is filed in the incorrect geographical region by mistake (see Figs 11, 12, 13).

The collection date can help us understand the extent of regional boundaries, especially when combined with contemporary maps created around the time of the collection date. Collection site names can change or disappear over time, usually due to human influence. This includes settlements, habitats, watercourses and waterbodies. One great example of this situation is the old pagoda near Amoy (i.e. BM000793283 and BM000996067). The pagoda itself was destroyed during the Cultural Revolution in 1968 (Kuiper 2017), but there is a picture of it, made in the early 1900s. The approximate location is marked with a memorial board. We can also find a mention of a place “Kulu” on Joseph Rock’s labels (i.e. BM000999987, BM000884297). Kulu is near Muli in the Muli Tibetan Autonomous County. There used to be three monasteries (Muli, Kulu and Waerdje). The monasteries were destroyed during the Cultural Revolution. The Muli monastery has been partially rebuilt, but the other two are still in ruins (China Trekking 2017).

Collection date is also helpful in terms of determining which romanisation system may have been used. As seen in the Georeferencing methodology section, the collection date can determine which georeferencing process we need to follow. We can also exclude romanisation systems from the “possible systems” list, when the collection date is known. For example, if a collection date is “1902”, we can be certain we cannot find names in pinyin on the label, as this was developed much later.

**Figure 14.**
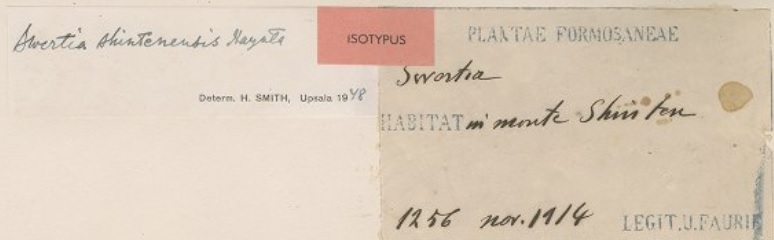
Specimen BM001014605 - The label reads: [Formosa] in monte Shin ten. Formosa is a previous name for Taiwan and Shin ten is the Japanese name for Xindian. The specimen was collected in 1914, when Taiwan was under Japanese rule.

**Figure 15.**
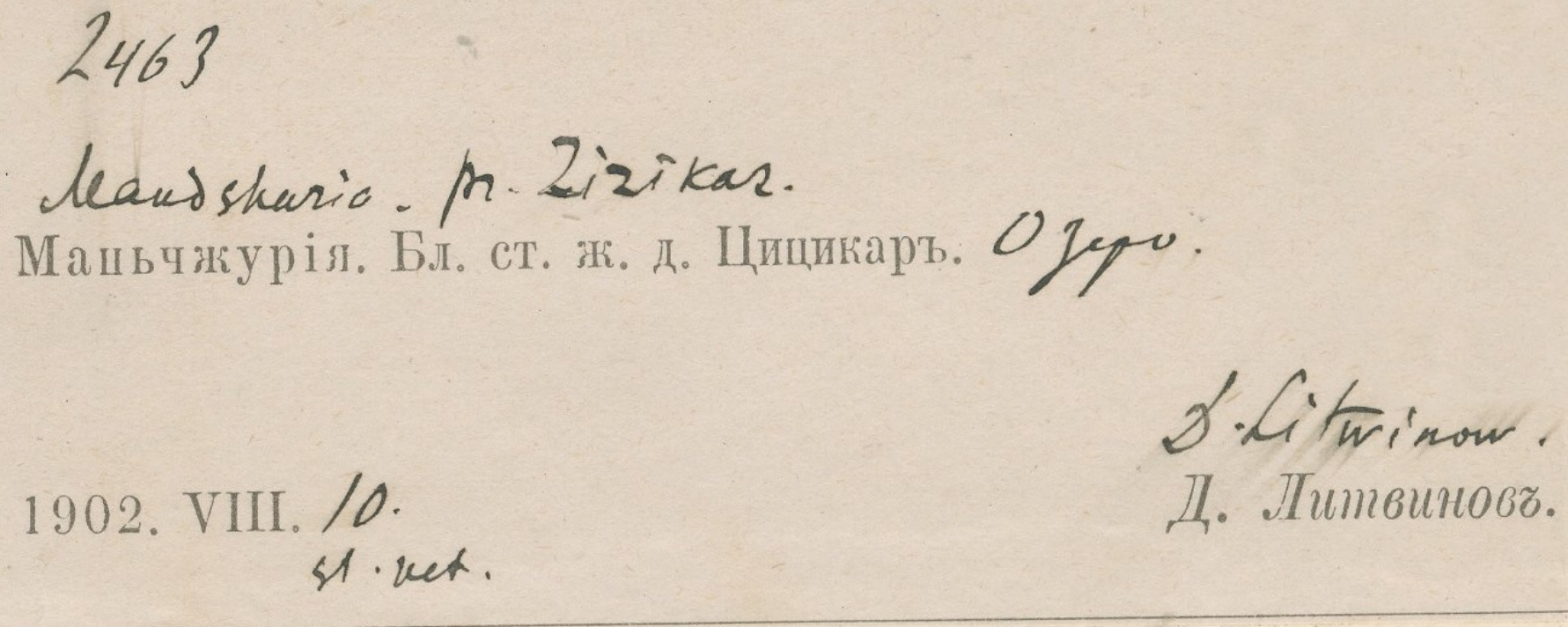
Specimen BM000570748 - The label reads Mandshuria. pr. Zizikar. (Маньчжурiя. Бл. ст. ж. д. Цицикаръ.) Zizikar (Цицикар(ъ)) is the Russian name for Qiqihar (齐齐哈尔). In 1902, when the collection was made, Qiqihar was under Russian influence, which persisted as Qiqihar was a central station on the Chinese Eastern Railway.

As previously mentioned in the Transcription Methodology, if a territory changed hands through the centuries, we can find the same locality with different names. In case a collector was in Taiwan or Manchuria during the Japanese occupation, it is highly likely that the name of the locality will be in Japanese (see BM001014605 on Fig. 14) or, in Manchuria’s case, it can also be in Russian (see BM000570748 on Fig. 15). In cases like this, sometimes we can find documents stating the name changes. If not, we need the locality’s name with the characters (as the characters will be the same), we just need the Chinese pinyin of the characters to get the Chinese name for the locality or we need to find a map that was issued during that time. After finding the locality on the map, we can get the coordinates and match it to a modern map to get the locality’s current name. The same applies for Manchuria, but the label can have Japanese, Chinese or Russian names on it (see examples in Figs 14, 15).

**Figure 16.**
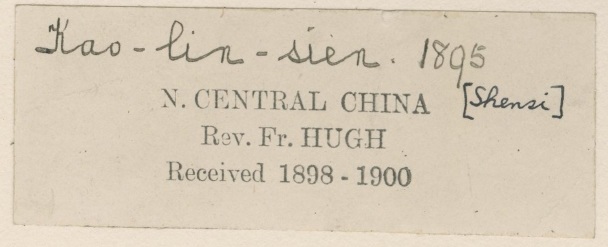
Specimen BM000630457 - “Schensi” (= Shaanxi) was transcribed “Schansi” (= Shanxi)

Some of the errors we encountered are likely to do with the lack of time which transcribers could spend on the labels. The easier-to-read fully printed labels (where all the information is printed and there is no handwriting on the label) were more likely to be transcribed than the handwritten ones, especially where the handwriting was not clear. The lack of time therefore resulted in partial transcription or mixed-up letters and numbers on occasions. Chinese is one of those languages, when a single letter mistake can drive the georeferencer into wrong directions (see Fig. 16).

Botanical sheets not only contain the plant, but can also contain numerous labels, writings, drawings and, quite often, multiple specimens mounted on the same sheet. One needs experience and time to confidently determine which label or other feature goes with which plant and to distinguish collection labels from determination labels. In this project, we needed curatorial help with less than 1% of the records. It is important to point out that this was a result of careful planning ahead. It was shown to us by curatorial staff where to find the books and publications to find the information we were looking for.

Being familiar with the languages most commonly appearing on the label and with commonly used words in that language, can also help reduce error rates. It happens occasionally that, due to the transcribers lack of familiarity with a language, they transcribe collectors as locality or other similar mistakes. As preparation for a project, a spreadsheet should be created for the most commonly occurring words, with translations or alternatives in any relevant languages. The most common languages can be determined through familiarisation with the location’s history and key events such as colonisation and historically important trading partners.

When a city or port played an important role in trade, different languages can have different names for the same place. The best example is Guangzhou (or Canton) which, due to its location, was an important trading city over the last 2,000 years. Many nations had their own name for it: either a differently spelled version of the original or an alternative name for another reason. The Wikipedia article for Guangzhou lists more than 10 names for the city (Wikipedia contributors 2020).

It is also important to mention that transcribing and georeferencing involve a range of skills and techniques which cannot all be measured. Experience, memory and local knowledge all contribute to getting the best results. This is particularly important when the data present challenges in legibility and when experience can recognise the idiosyncrasies of particular collectors.

In the NHMUK collection, plants which were collected by Nathaniel Wallich are a good example of how previous experience could have helped in the transcription and georeferencing. The collector was transcribed correctly, but Wallich’s catalogue numbers were incorrectly transcribed as collection numbers. We can also use Wallich’s catalogue numbers, as his labels were frequently missing collection site information, but the catalogue is available online, where the missing collection information can be found using the catalogue numbers.

Transcription and georeferencing are not easy tasks, especially when the collection area’s main language is so different from the language of the country housing the collection. By using a detailed methodology and a careful, structured approach, the quality of the transcription data can be increased, providing a stronger baseline dataset for better, faster and therefore more cost-effective georeferencing.

### Lessons learned

**Transcription**

- To get transcription with the required quality, adequate time and training need to be provided to the transcribers.
- Due to the complexity of herbarium sheets, the transcription process needs to be closely monitored by curators, providing help where needed.
- Transcription guidelines are needed for questionable labels and label information and to support transcription in other languages, for example, to provide keywords.

**Georeferencing**

- When dealing with collections with non-European languages, georeferencing (as well as transcription) needs language and transliteration guidelines.
- Standards for locality georeferencing need to be implemented, as well as standards for recording missing and illegible information.

**Project management**

- The size of the collection should not be underestimated. Providing the necessary amount of time is essential. Generally, a quality over quantity approach leads to better results for transcription and georeferencing, reducing future re-work. It is challenging to estimate the time and resources required for a compex georeferencing project, especially in externally-funded projects. We would recommend agreeing a per record time limit for georeferencing and recording which could not be resolved within this limit. Some specimens may be more easily georeferenced once others have been done or could be georeferenced if there is time remaining at the end of a project.

**Data Sharing**

- It is hard for us to share georeferencing protocol information, including data sources that were used for individual records. We inconsistently recorded this information in our collections management system and when it is recorded, it is not always done in a structured way that can be easily shared on our data portal. This reduces the re-usability of potentially useful data that other collections could use.

### Conclusions and possible future work

For this project, collection size, project duration and the need for re-work on previous transcription meant that we could not spend as much time on georeferencing as originally planned. There are specific areas which need more time to determine and verify specific localities, for example, the “Lien chau” river in the vicinity of Guangzhou. In the future, we now know that we need to approach other countries / geographical regions with caution, as the transcription quality could be lower than expected. It would be a useful to build a database of collector’s publications prior to starting a future project, especially if certain collectors are very frequent in a collection. Many publications are available in the institutional libraries, but they are more useful in a machine- and human-readable digital format, where one can search the text to find the information needed. Many of these publications are available online for free or at least partially. We found it useful to have these publications ready when we started to work the collection, to speed up the transcription process.

This project was useful in terms of understanding that the quality of the initial transcription project and the data from it, was not as expected. We cannot set a tight schedule on transcription projects, as this will lead to poor quality data. Providing sufficient time and training and working closely with a curator, can help to reduce transcription errors.

Accurate transcription can help us make statistical analyses on the collection, for example, what areas were most collected; or which collectors provided how many specimens. We can concentrate on more prolific collectors first, mapping their localities, since they likely collected for a longer period covering more areas. Mapping those localities could help us to map the rest of the collection. This is especially true if those collectors used spellings that are not in use anymore.

We hope that the collated dataset about the collectors and the methodology provided in this paper, provide a helpful guide for future projects working on East Asian collections, as well as a starting point for other regions, based on non-Latin alphabets.

## Terms of Reference

**GPI (Global Plants Initiative)**: “The GPI seeks to digitize and make available plant type specimens and other holdings used by botanists every day. Partners include more than 300 institutions in more than 70 countries. JSTOR facilitates this initiative by providing production, platform, technical, and promotional support to the participating Global Plants Initiative partners.” (source: https://www.jstor.org)

**Locality**: Also referred to as an *exact locality* or *collection site*, is the exact location where someone collected a specimen, ideally referring to one point on the map. Country, province or “mountain range” are more broad locality descriptions. Exact locality would be like “on the East side of the Amoy pagoda” or “Karo La Pass”.

**NHMUK**: The Natural History Museum, London.

**Protologue**: In taxonomy, all the original material associated with a newly published name, comprising its description or diagnosis and any of a number of other elements such as illustrations, synonymy etc.

**Romanisation**: In linguistics, it is the conversion of writing from a different writing system to the Roman (Latin) script or a system for doing so.

**Transliteration**: A type of conversion of a text from one script to another that involves swapping letters in predictable ways

## Specimens cited

All specimens cited as examples within the text and figure captions are provided in Table 5.

## Supplementary Material

B7ADF68B-1320-5432-950D-26277F2FF672 10.3897/BDJ.8.e50503.suppl1 Supplementary material 1 Collectors Data type collector names Brief description List of collectors for the specimens transcribed in this project along with links, where they exist or were able to be created, to their Wikidata and JSTOR Global Plants pages. File: oo_375167.csv https://binary.pensoft.net/file/375167 Krisztina Lohonya

1F7516BE-9A18-55BE-8999-0D21DF037F67 10.3897/BDJ.8.e50503.suppl2 Supplementary material 2 Province distribution Data type specimen distributions Brief description Summaries of specimen distribution by Chinese provinces. File: oo_375169.csv https://binary.pensoft.net/file/375169 Krisztina Lohonya, Laurence Livermore

7F36CF06-7C05-5963-9876-DCA4B1BBE365 10.3897/BDJ.8.e50503.suppl3 Supplementary material 3 Erroneous country distributions Data type distribution data File: oo_375139.xlsx https://binary.pensoft.net/file/375139 Krisztina Lohonya, Laurence Livermore

566B0B8A-B518-50F8-9270-09B62827B6A3 10.3897/BDJ.8.e50503.suppl4 Supplementary material 4 Spelling variations of Chinese provinces Data type spellings, provincesspellings, provinces Brief description Reference data for spelling variations of Chinese provinces. File: oo_375171.csv https://binary.pensoft.net/file/375171 Krisztina Lohonya

30456F8F-444C-5708-A98C-D530886FD376 10.3897/BDJ.8.e50503.suppl5 Supplementary material 5 Frequently occuring localities Data type locality information, coordinates, geography Brief description Frequently occurring georeferenced specimen localities with latitude, longitude, administrative, province, country and reference. File: oo_375172.csv https://binary.pensoft.net/file/375172 Krisztina Lohona

1DF0BA3D-0BAF-5379-B218-E04B617F2826 10.3897/BDJ.8.e50503.suppl6 Supplementary material 6 Other data sources Data type links Brief description Other data sources not directly cited in the text, but used to georeference, determine authors or cross-referencing collecting information. File: oo_375173.csv https://binary.pensoft.net/file/375173 Krisztina Lohonya

## Figures and Tables

**Table 1.** Example of different romanisation systems based on Qinghai province (青海省):

**Romanisation system**	**Romanised name**
Modern (pinyin)	Qinghai
Wade-Giles	Ch'ing-hai
Portuguese	Chinghai
German	Tschinghai
French	Ts'ing-hai

**Table 2.** Abbreviations commonly used in natural history collections when information is missing or unknown. The ones we used are indicated with an asterix.

**Collector**	**Collection number**	**Collection date**	**Locality**
Anonymous*	s.n.*	sino dato	sine loco
Anon.		sin. dat.*	sin. loc.*
		sd	s.l.
		s.d.	

**Table 3.** Spelling differences between the romanisation systems.

**Pinyin**	**Wade-Giles**	**EFEO**
cha	ch'a	tch'a
zha	cha	tcha

**Table 4.** Generic localities commonly appearing on labels.

**English**	**Chinese**	**French**	**German**	**Latin**
Mountain	shan	montagne	berg	mons
(Mountain) ridge	ling	Crête de la montagne	Bergrücken	Iugum (demittere)
Valley	gu	vallée	Tal	valley
River	he/jiang/chuan	rivière	Fluss	flumen
Road	tu	route	Straße	via
City	shi	ville	Stadt	urbs
Village	cun	village	Dorf	village
Gorge	gu	gorge	Schlucht	Torrentis Arnon
Forest	lin	forêt (or as often seen on label "bois de...")	Wald	silva

**Table 5.** Specimens cited in the text by order of appearance. All specimens are from the Natural History Museum, London (NHMUK) collections.

**Catalogue Number**	**Scientific Name**	**Permanent Object URL**
BM001125208	*Leontopodium villosum* Hand.-Mazz.	https://data.nhm.ac.uk/object/91dda2f2-c548-4230-876b-9159c34a9d8a/1579737600000
BM000796373	*Cycas multiovula* D.Yue Wang	https://data.nhm.ac.uk/object/eca72c84-ffb2-4b5d-89ce-5e737256846a/1579737600000
BM001047709	*Cycas segmentifida* D.Yue Wang & C.Y.Deng	https://data.nhm.ac.uk/object/ed93aa21-a830-431e-ab69-54afae9360f8/1579737600000
BM001044167	*Adiantum capillus-junonis* Rupr.	https://data.nhm.ac.uk/object/af65d806-7378-438b-b8d3-5e03fb08a065/1579737600000
BM001066167	*Elaphoglossum fuscopunctatum* Christ	https://data.nhm.ac.uk/object/ff5c49e6-845f-4689-bcc6-311d21ef27a4/1579737600000
BM001014605	*Swertia shintenensis* Hayata	https://data.nhm.ac.uk/object/7e1bdd32-c383-49b1-9334-476030283eb9/1579737600000
BM000959201	*Thamnocalamus spathaceus* (Franch.) C.D.Chu & C.S.Chao	https://data.nhm.ac.uk/object/1b033c71-f4ec-449d-ab41-4d1f7339e025/1579737600000
BM000554710	*Clematis hexapetala* Pall.	https://data.nhm.ac.uk/object/56869d37-bda7-4bb0-a0f4-c1f567a8c81c/1579737600000
BM000559554	*Thalictrum aquilegifolium* L.	https://data.nhm.ac.uk/object/4db7e422-2276-4006-b8c0-54212bece419/1579737600000
BM000996109	Ptilostemon chamaepeuce var. cyprius Greuter	https://data.nhm.ac.uk/object/fee20cb8-a164-4c5e-bbf8-4db1743d960d/1579737600000
BM000570748	*Potamogeton manchuriensis* (Benn) Benn	https://data.nhm.ac.uk/object/52c807ee-463c-4900-b90a-1e9495ba8935/1579737600000
BM000630457	*Poa faberi* Rendle	https://data.nhm.ac.uk/object/99499af8-f0af-42b8-9fa7-82b1a5c13ce8/1579737600000
BM000793283	*Abelia chinensis* R.Br.	https://data.nhm.ac.uk/object/d3a0b717-421d-4e29-8b99-42c78d350789/1579737600000
BM000996067	*Echinops dahuricus* DC.	https://data.nhm.ac.uk/object/9291a56a-7694-4331-8512-64168149cdcf/1579737600000
BM000999987	*Primula rockii* W.W.Sm.	https://data.nhm.ac.uk/object/7c310a34-ea9c-454e-92e7-49f1ebd24af6/1579737600000
BM000884297	*Acer pentaphyllum* Diels	https://data.nhm.ac.uk/object/c439a600-436c-4879-9b1b-c1b4fb41e404/1579737600000
